# A validation of the religious and spiritual struggles scale among young people living with HIV in Zimbabwe: Mokken scale analysis and exploratory factor analysis

**DOI:** 10.3389/fpsyg.2023.1051455

**Published:** 2023-04-18

**Authors:** Ursula Wüthrich-Grossenbacher, Abigail Mutsinze, Ursula Wolf, Charles Chiedza Maponga, Nicholas Midzi, Masceline Jenipher Mutsaka-Makuvaza, Sonja Merten

**Affiliations:** ^1^Centre for African Studies, Faculty of Humanities and Social Sciences, University of Basel, Basel, Switzerland; ^2^Zvandiri, Harare, Zimbabwe; ^3^Institute of Complementary and Integrative Medicine, School of Medicine, University of Bern, Bern, Switzerland; ^4^School of Pharmacy, University of Zimbabwe, Harare, Zimbabwe; ^5^School of Pharmacy and Pharmaceutical Sciences, University at Buffalo, Buffalo, NY, United States; ^6^Ministry of Health and Child Care, National Institute of Health Research, Harare, Zimbabwe; ^7^Epidemiology and Public Health, Swiss Tropical and Public Health Institute (Swiss TPH), Basel, Switzerland; ^8^Faculty of Medicine, University of Basel, Basel, Switzerland

**Keywords:** religious and spiritual struggles scale, HIV, adolescents living with HIV, Zimbabwe, validation, spiritual struggles, religion

## Abstract

**Introduction:**

Religious/spiritual convictions and practices can influence health- and treatment-seeking behavior, but only few measures of religiousness or spirituality have been validated and used outside of the US. The Religious and Spiritual Struggles scale (RSS) measures internal and external conflict with religion and spirituality and has been validated mainly in different high-income contexts. The aim of this study was the validation of the RSS in the Zimbabwean context and among young people living with human immunodeficiency virus (YPLHIV) aged 14–24.

**Methods:**

Data collection with an Open Data Kit (ODK) questionnaire with 804 respondents took place in 2021. The validation was performed by confirmatory factor analysis (CFA), using statistical equation modeling (SEM), and Mokken scale analysis (MSA). After the low confirmability of the original scale sub-dimensions exploratory factor analysis (EFA) was applied.

**Results:**

The EFA resulted in four new sub-domains that were different from the original six domains in the RSS but culturally more relevant. The new sub-domains are significantly related to health.

**Discussion:**

The findings support the validity and relevance of the RSS and the new sub-domains in this context. As our study was limited to YPLHIV, further validation of the RSS among different population groups and contexts in the sub-Saharan region is encouraged.

## Introduction

Religious/spiritual (R/S) struggles are defined as tension and conflict about R/S matters within oneself, with others, and with the supernatural (Stauner et al., 2016). Numerous studies, mainly within the field of the psychology of religion, have indicated that R/S struggles relate negatively to health and wellbeing (Ellison et al., 2013; Bockrath et al., 2021). Exline et al. illustrate that R/S struggles are linked with emotional distress, including depressive symptoms, anxiety, and signs of other emotional disorders, as well as suicidal ideation (Exline et al., 2014). Furthermore, the findings of a recent meta-analysis of longitudinal studies indicated that R/S struggles significantly predicted increases in negative psychological adjustment (Bockrath et al., 2021). These findings highlight the clinical relevance of R/S struggles. As adolescents search for meaning, significance, and identity, R/S struggles may be prevalent among them (Parker et al., 2022). They can include struggles related to parents/caregivers (incongruent beliefs), peers (fear of losing friendships, belonging), and identity development (deciding one’s own religious beliefs) (Homolka, 2017). Hence, this study explores the prevalence and influence of R/S struggles among our study population of young people living with human immunodeficiency virus (YPLHIV) in Zimbabwe. Often, R/S struggles are measured by the validated Religious and Spiritual Struggles scale (RSS) (Exline et al., 2014; Esperandio et al., 2022). By spirituality, the developers of the RSS refer to a search for the sacred—elements of life that are seen as manifestations of the divine, transcendent, or ultimate, either inside or outside of a specific religious context. For them, religion takes place in the larger context of established institutions and structures that aim to facilitate spirituality (Exline et al., 2014). Developing sound scientific tools that measure the complexity of religion and spirituality remains a challenge (Hall et al., 2008; MacDonald et al., 2015; Finke and Bader, 2020). According to Hill and Pargament, there are three general measurement issues: theoretical considerations, psychometric issues, and sample representativeness (Peter C. Hill and Kenneth I. Hill and Pargament, 2017). We opt for the RSS, because it meets Hill’s criteria of “good” measures in terms of theoretical grounding, sample representativeness, reliability, and validity (Hill, 2013). The RSS provides a comprehensive, reliable, concise measurement of multiple domains of R/S struggles (Magyar-Russell, 2021). It assesses six domains of R/S struggles. Two domains focus on beliefs about supernatural agents, divine struggles (negative emotions related to God or regarding one’s relationship with God), and demonic struggles (concerns that evil forces/Satan influence one’s life). Three domains emphasize intrapersonal struggles with an inward focus on one’s own thoughts or actions. The three domains of intrapersonal struggles are moral struggles (personal wrestling with attempts to follow moral principles), doubt-related struggles (being troubled by doubts or questions about one’s R/S beliefs), and struggles around ultimate meaning (being concerned about a perceived lack of deep meaning in life). One domain focuses on interpersonal struggles (negative emotions, experiences, and conflicts with religious people or institutions) (Exline et al., 2014). Hence, compared to other measures of R/S struggles—i.e., the Religious Coping scale (Pargament et al., 2011), the Attitudes Toward God scale (Wood et al., 2010), and the Religious Doubt scale (Altemeyer, 1988)—we find that the RSS is the most comprehensive measure covering all domains of R/S struggles.

Furthermore, the RSS has already been validated in different languages (e.g., Portuguese, Persian, Polish, and Czech) and different cultural and religious contexts, namely, in Brazil (Esperandio et al., 2022), Iran (Ebrahimi Jamarani et al., 2022), Poland (Zarzycka et al., 2018), among people with Jewish (Abu-Raiya et al., 2016) and Muslim (Abu-Raiya et al., 2015) religion in Israel, and in the Czech Republic (Janu et al., n.d.). This is important, because most R/S-measuring tools (including the RSS) have been developed in the Judeo-Christian Western context (Büssing, 2019). Hill and Pargament point to the importance of knowing the local culture and religious context to better understand the phenomenon of interest in the given culture. This must be the condition for making the decision as to whether the underlying construct and its measure should be used (Hill and Pargament, 2017). In the context of Africa, a recent exploratory qualitative study in six eastern and southern African countries has shown that medical pluralism, manifesting across traditional, faith-based, and biomedical health worlds, contributes to delays and interruptions of care along the HIV cascade and mistrust between health providers. Thus, it is argued that the role of sociocultural beliefs necessitates the adoption of culture-sensitive approaches, intervention designs, and policy reforms (Moshabela et al., 2017). We hope that the validation of the RSS in the Zimbabwean context will show whether it is such a culture-sensitive approach see (Table 1).

**Table 1:** Participants’ sociodemographic characteristics in frequency and percentage.

Variable	Frequency overall number: 802	Percentage
*Age*		
Below 18	481	59.98
Between 18–24	321	40.02
*Gender*		
Male	308	38.4
Female	492	61.35
Other	2	0.25
*Location*		
Rural	300	37.41
Peri-urban	105	13.09
Urban	386	48.13
Missing	11	1.37
*Earning in US Dollars/month for 18–24 years old*		
Less than 10	161	50.2
Between 10–20	53	16.5
Less than 25	62	19.3
Between 25–50	29	9.0
Between 50–150	6	1.9
Over 150	10	3.1
*Education*		
Never went to school	9	1.12
Below Grade 7	48	5.99
Grade 7	161	20.07
Form 1–4 (secondary)	524	65.34
A Level (university entry exam)	40	4.99
Tertiary level (post-secondary)	20	2.49
*Civil status*		
Single	525	65.46
Boyfriend/Girlfriend	200	24.94
Live-in-partner	16	2
Married	51	6.36
Divorced	9	1.12
Widowed	1	0.12

RSS validation study among young people living with HIV in Zimbabwe in 2021.

### The Zimbabwean context

With a prevalence of about 12.9% among adults aged from 15 to 49 years, HIV continues to have a major impact on morbidity and mortality in Zimbabwe (2020). According to UNICEF, in Zimbabwe, one-third of all new HIV infections are in adolescents and young people (15-24 yrs), and adolescent and young females are twice as likely to contract HIV than males. Stigma prevents adolescents accessing HIV/sexual reproductive health services, and there is limited space for the meaningful participation of adolescents in decision-making.^1^ Attrition from antiretroviral therapy (ART) remains a serious challenge (Makurumidze et al., 2020). Thus, new methods of care are needed to address risk factors to the adherence to ART that have not been considered sufficiently. To our knowledge, the role of R/S struggles for YPLHIV in Zimbabwe has not been investigated. Hence, the findings of this study might generate new insights on new and culturally sensitive approaches of support for YPLHIV.

In Zimbabwe, R/S plays a major role in daily life, substantially influencing people’s health-seeking behavior (Mutambara et al., 2021). The findings of a scoping literature review about the influence of R/S on HIV in Zimbabwe describe that R/S aspects, including traditional beliefs and practices, play an important role for most people and can be barriers or facilitators for health and wellbeing Wuthrich Grossenbacher et al. (2021). Many Zimbabweans conceive and practice healing holistically, embracing not only the physical conditions but also the spiritual, psycho-emotional, social, and ecological dimensions (Tabona Shoko, 2015). Most Zimbabweans would call themselves ‘Christian’. However, the Christian faith is practiced diversely. There are many different Christian churches and groups. Since around 2009, Zimbabwe has witnessed a surge of Christian preachers who call themselves prophets or prophetesses. These prophets and prophetesses claim to be mediators between God and ordinary people, and they profess to work miracles such as healings (Chitando et al., 2013). Zimbabweans may be members of different churches, followers of different prophets, or swap from one to the other. Many Zimbabweans readily combine Christian belief and traditional practices. Moreover, as Chavunduka explains, there are many similarities in the medical practice of Christian prophets and traditional healers (Chavunduka, 1994). Both tend to concentrate on the social and psychological cause of illness. The difference is the medium through whom the knowledge of the diagnosis is revealed. For the traditional healer, the source of revelation is the spirits, and to the Christian prophets, the source of revelation is the Holy Spirit. Chavunduka describes that most Shona and Ndebele people of Zimbabwe believe that people do not die, but they merely pass on from this world to the world of spirits. To become accepted in the spirit world, the deceased person must have left behind children, and relatives must organize a ritual to hand the spirit of the deceased over to the other ancestral spirits. Before the new spirit joins other spirits, it is considered unpredictable and dangerous. Hence, an angry ancestor may withdraw blessings with eventual negative impacts on health, among others (Chavunduka, 1994). If an illness becomes more severe, many people therefore seek help at a medical clinic and a Christian prophet or a traditional healer. Especially if Western medicine cannot give the desired result, an illness that might have been considered “natural” at first might then be interpreted as supranatural or of social origin.

This rich religious and cultural background could be a challenge for YPLHIV. Health decisions and religious and traditional practices are not individual decisions in the Zimbabwean context. YPLHIV in Zimbabwe must adhere to the rules and customs of their family. The potentially fatal consequences of this was described in a recent Zvandiri study, in which it was demonstrated that up to 73% of deaths of YPLHIV were due to the cessation of ART sanctioned by caregivers, probably motivated by faith healing. Mavhu et al. (2020) In other words, while YPLHIV share the experience of searching for identity, significance, and meaning together with other youth, their health is more vulnerable, which increases their dependency on family/caregivers and policymakers. Wanting to belong, dependency on others, and, at the same time, the fear of stigma and exclusion make for a dangerous mixture. YPLHIV know that possible incongruent religious and traditional beliefs and practices would threaten the very social fabric they so heavily depend on. Thus, for YPLHIV in Zimbabwe, the scope for R/S coercion is real. This could seriously impact their wellbeing and health. Hence, it is important to have a culture-sensitive tool to measure the influence and impact of R/S struggles on the wellbeing of YPLHIV. To our knowledge, the RSS has not been used in Southern Africa before. Validation of the scale and its subscales will provide a tool for the improved understanding and culturally significant, comprehensive care of YPLHIV.

### Study objectives

- To confirm/refute the RSS scale and its subdimensions among adolescents in Zimbabwe by confirmatory factor analysis (CFA) using structural equation modeling (SEM)
- In case of refutation: To assess the unidimensionality of the RSS or identify subscales by using Mokken scale analysis (MSA)
- To identify new, culturally valid subdomains of RSS using Exploratory Factor Analysis (EFA)
- To verify the internal consistency and reliability of the entire RSS and subdomains by using Cronbach’s alpha
- To assure the conformity of the identified subscales by external scale item validation

## Methods

The validation of the RSS is part of a bigger mixed study about the role of religion/spirituality for YPLHIV approved by the Medical Research Council Zimbabwe (MRZ/A/2701).

### Methods of quantitative part (mother study)

The quantitative part, with 802 HIV program clients (Zvandiri cohort)^2^ of both sexes from urban, peri-urban, and rural areas, contained the following baseline data: Demographic factors (e.g., age, location, education, civil status, income—these factors were used to describe the study population and possible effect modifiers), health parameters (viral load, opportunistic infections, self-rated health, mental health), questions regarding the influence of COVID-19, questions regarding risk behavior (including the experience of violence), religious affiliation, types of healers, and types of traditional medicines and practices involved in the patients’ lives. The following R/S measures have all been validated in different cultural contexts: Belief in Action scale, with 10 items assessing religious involvement (organizational and non-organizational religious activities and the degree of personal devotion or commitment to one’s religious faith) (Koenig et al., 2015); a shortened Religious Coping Index, with seven items measuring religious coping with life stressors (three items of positive religious coping, three items of negative religious coping, and one general question regarding the degree of involvement of religion/belief in coping with stressful situations) (Pargament et al., 2011); and the RSS (Bifactor Models of Religious and Spiritual Struggles are distinct from Religiousness and Distress) (Stauner et al., 2016). The primary endpoint of the entire study (=mother study) was the mental health status, measured with the validated 14-item Shona Symptom Questionnaire (Patel et al., 1997). The secondary endpoint was the viral load. To get a statistical power of 80% for this secondary endpoint, we aimed at a minimum of 800 participants.

Together with the entire questionnaire, the RSS was translated by a professional into the two major local languages, Shona and Ndebele. A transdisciplinary team consisting of the Zvandiri research team and the data collectors from the community checked and revised the translation for meaning and coherence during the piloting phase. The data collection team was recruited by Zvandiri among their community adolescent treatment supporters (CATS). After intensive training of all the data collectors and a successful pilot study in May 2021, the quantitative data collection with the ODK questionnaire in Shona, Ndebele, and English took place from July to October 2021. Due to the COVID-19 pandemic, most of the questionnaires had to be administered by phone or ODK self-administration *via* a public link. Most questionnaires were administered in Shona. Only few participants opted for the English or Ndebele version. Consent was given verbally and recorded. In the case of minors, parental consent was asked first, and then the child assented. The administration of the entire questionnaire took up to 60 min.

### Study population

The 802 participants (aged 14–24) for the quantitative study part belonged to the cohort of Zvandiri’s peer support program in Zimbabwe. Participants needed to be between 14 and 24 years of age, HIV positive, on ART, and have a current viral load result (not older than 12 months). They were selected from Ministry of Health and Child Care (MoHCC) facilities in seven districts. The facilities were purposefully chosen to ensure a good balance of location (rural, urban, peri-urban), religious environment (Apostolic sects, main churches, traditional), languages and cultures (Shona, Ndebele), and facility structure (difference in user fees).

### Validation of the RSS

The RSS translation was checked and revised by the data collection team during the piloting phase. However, at this point, unfortunately, there was not yet a back-translation conducted by someone outside the research team. The RSS was administered by phone or ODK self-administration *via* a public link.

The 26 questions of the RSS started with “Over the past few months how often have you encountered the following,” and the possible answers had a five-point Likert scale: 1: not at all/does not apply; 2: a little bit; 3: somewhat; 4: quite a bit; and 5: a great deal.

The validation of RSS was conducted by CFA^3^ using SEM^4^(“Structural Equation Modeling (SEM) | Stata,” n.d.). This was followed by an MSA (Hardouin et al., 2011a). For the MSA, we dichotomized the data into considerable and strong R/S struggles (Likert scale 3–5) and low or no R/S struggles (Likert scale 1–2). We then identified four relevant new dimensions with the result of EFA. These dimensions were further validated by measuring their affiliation with four questions relating to religion from the bigger questionnaire.

### Data analysis using Stata

Interviewers and public link users entered the answers into ODK using tablets or phones. The statistical software STATA version 17.0 (Stata Corp, College Station, TX, United States) was used to analyze the data. As most questionnaires were administered in Shona and only very few participants used the English or Ndeble version, the validation was performed with the Shona version of the RSS.

### Kaiser–Meyer–Olkin (KMO) and Bartlett’s test of sphericity

We first checked whether the variables of the RSS met the conditions for a confirmatory factor analysis (CFA). To test the sampling adequacy for the RSS, we conducted a KMO test. KMO values closer to 1.0 are considered ideal. It is commonly agreed that KMO values of at least 0.80 show a strong partial correlation and are good enough for factor analysis to commence (KMO and Bartlett’s test of sphericity, 2020). The following Bartlett’s test of sphericity checked the relation between the variables. A significant statistical test shows that the correlation matrix is not an identity matrix and is thus ideal for CFA.

### Structural equation modeling (SEM) for confirmatory factor analysis

SEM was used to identify relations between the domains and items with the latent construct of spiritual struggles. Some authors suggest sample sizes relative to the number of parameters being estimated. Ratios from 5:1 up to 20:1 have been proposed for SEM to work properly (Huber, 2014).

### Mokken scale analysis (MSA)

To address non-normal distribution of the item responses, we dichotomized answers and conducted MSA with a non-parametric item response theory-based approach (Stochl et al., 2012). MSA is a method to assess the psychometric quality of questionnaires and their individual items. It assesses the three fundamental assumptions of item response theory: unidimensionality, monotonicity, and local independence (Hardouin et al., 2011b). Unidimensionality is given when all items of a scale or sub-scale measure the same latent trait (Ziegler and Hagemann, 2015). The Loevinger’s scalability coefficients permit the assortment of items that measure the same latent trait in the Mokken scale from an item group. A Mokken scale is considered a weak scale when 0.3 ≤ H < 0.4, a medium scale when 0.4 ≤ H < 0.5, and a strong scale when H > = 0.5 (Crişan et al., 2020).

### Exploratory factor analysis (EFA)

After refutation of the original sub-dimensions of the RSS scale, we identified new, culturally relevant sub-dimensions by applying EFA. First, we used Stata’s factor test to assess the appropriateness of a factor analysis. The factor test performs Bartlett’s test for sphericity and calculates the Kaiser–Meyer–Olkin measure (KMO) of sampling adequacy. A Bartlett’s test of sphericity with a significant value of *p* of <0.05 and KMO of at least 0.5 is needed to proceed with EFA^6^.

The sub-domains of the 26-item RSS were identified by using the principal component factor analysis. We retained factors with eigenvalue ≥1. We used orthogonal varimax rotation and sorting for easier interpretation. Several items were loaded on more than one factor. Stevens recommends retaining factor loadings of 0.400 or greater (Stevens, 2009). The items were considered to belong to the factor under which they had the highest loading value and loadings were above 0.4. We used Cronbach’s alpha to determine the internal consistency reliability of the subdomains and overall RSS. Generally, Cronbach’s alpha of 0.7 and above is considered reliable (Taber, 2018). The resulting dimensions with corresponding items were compared with the subdomains identified by MSA.

### External validation of subscale

The subdomains identified by EFA were further evaluated by descriptive statistic measures. The subdomains were compared with the results of the following variables in the overall questionnaire: “God is 1^st^ priority in life,” “more than one religious affiliation,” “time spent for religious activities,” and “believe that God exists without doubt.”

In consideration of the local cultural and religious context, the detailed description of the sub-domains was conducted by analyzing the relationship between the domain items and the following variables of the bigger questionnaire: Belief about God, Belief about Spirits, Religious Affiliation, Highest Priority, Religious Coping, Extent to which Religion Belief is used to understand and deal with Stress, Conformity with Religious Teaching, Ever Stopped antiretroviral therapy for Religious Reasons, Number of Herbal Supplements use, Religious Reasons against Cervical Cancer Screening, Religious Reasons against the use of Western Medicine, and Alternative (other) Explanations for HIV/AIDS.

## Results

### Participants’ sociodemographic characteristics

The sociodemographic characteristics of the study participants can be seen in Table 1.

#### Participants’ RSS results

A total of 16% of the participants with an average score ≥ 3 were considered to experience considerable and high R/S struggles

### Validation of RSS

The result of the KMO for the RSS was above 0.95. Bartlett’s test of sphericity was below the 0.05 significance level. This allowed us to proceed with the confirmatory factor analysis.

### Confirmatory factor analysis with SEM

SEM did not converge and did not show a coherent result. The domains “Doubt” and “Interpersonal” were prominent, but “Interpersonal” had no significant value. It might be that a bigger sample size is needed to get a sound result.

### Mokken scale analysis

The distribution of the response categories was non-normal, and it was positively skewed (0.2). We therefore applied Mokken scale analysis to assess the one-dimensionality and scalability of the RSS.

The MSA of RSS with 5-point Likert scales only identified one dimension, excluding item 1. After dichotomizing the data, we carried out another MSA, resulting in four dimensions and four excluded items. The first dimension had 10 items, the second 6, the fourth 4, and the last only 2 items. All the dimensions had items from different original domains, except the second dimension, where four out of six items belonged to the original Divine sub-domain.

To verify whether this was a translation issue, the items were back-translated into English by a person outside the research team. Out of the four items excluded by the MSA, three had translation issues. Because we identified four dimensions that were different from the original RSS, we decided to conduct an EFA for further exploration.

### Identification and validation of new sub-domains (=Factors)

We then conducted an EFA using a principal component factor analysis. The four factors generated by the EFA gave a coherent picture of a specific religious and spiritual struggle (alienation, confusion, doubt/conflict, and religious zeal as can be seen in Table 2). The items were evenly distributed with no exclusion, and most of the items corresponded to an MAS domain as shown in Table 3.

**Table 2:** Factors resulting from the principal component factor analysis.

Factor	Items (loading value in brackets)
Alienation	I felt as if God had let me down. (0.73)
	I was not happy with the way religious activities are carried out. (0.69)
	I felt like God was punishing me over something. (0.65)
	I found myself questioning/confused about religious/spiritual matters. (0.62)
	I felt pained by the way the believers treated me. (0.61)
	I felt like God has forsaken me. (0.57)
	People do look down upon me because of my beliefs in religion/spirituality. (0.48)
Confusion	Failing to know whether my ways of living are accepted by what is written in the Bible/spiritually, am I doing good or bad? (0.7)
	Feeling like my life has no deeper meaning. (0.63)
	The thought of not believing and questioning Religion/Spirituality worried me a lot. (0.62)
	I felt misunderstood and disowned by those I attend church with or those at my spiritual gathering. (0.57)
	I felt like the devil or evil spirits were tempting me to do bad. (0.51)
	I was angry with God. (0.51)
	Failing to distinguish between what is good and what is wrong. (0.51)
Doubt/Conflict	I asked whether my existence has any difference/meaning on this Earth. (0.77)
	I felt conflicted between what I wanted to do and what I know is regarded as the right way of doing things. (0.7)
	I felt as if all my troubles (trials and tribulations) are a result of the works of the devil and evil spirits. (0.53)
	Debating whether what I believe in is correct, that is religion or spirituality wise. (0.51)
	I felt like the devil and evil spirits were working to get me. (0.49)
	I asked whether my life had a purpose or was worth it. (0.48)
Religious zeal	I felt attacked by the devil or by evil spirits. (0.72)
	I found myself guilty of living in sin rather than what I know is right. (0.65)
	I did not get along with other people because of my views regarding religion and spirituality. (0.65)
	Asking myself whether God truly loves me. (0.62)
	Struggling to understand or believe in the word of God or in spirits. (0.44)
	Asking myself if there is a reason to live. (0.42)

Items were put to the factor under which they had the highest loading value. RSS validation study among young people living with HIV in Zimbabwe in 2021.

**Table 3:** Comparison of EFA factors and MSA sub-domains.

Variable	Factor 1 Alienation binary (items’ score average > 3)	Factor 2 Confusion binary (items’ score average > 3)	Factor 3 Conflict/ Doubt binary (items’ score average > 3)	Factor 4 Religious zeal binary (items’ score average > 3)
Overlap with Mokken	4 out of 7 items	5 out of 7 items	4 out of 6 items	4 out of 6 items

RSS validation study among young people living with HIV in Zimbabwe in 2021.

The external validation of the four new sub-domains was performed with following variables of the bigger questionnaire: “God is 1st priority in life,” “more than one religious affiliation,” “time spent for religious activities,” and “believe that God exists without doubt.” Affiliations of these variables with the new sub-domains were checked with regression analysis and compared. The results can be seen in Table 4. Underlined values signify the highest value, and values in italic are the lowest values. The Alienation factor is confirmed by the lowest value in having God as the priority. The Confusion factor is confirmed by the highest value in more than one religious affiliation. The Doubt factor is confirmed by the lowest value in the doubtless belief in the existence of God. The Religious Zeal factor is confirmed by the highest value for the time spent with religious activities.

The relevance of the four factors for the participants’ self-rated health was then checked with ordered logistic regression controlling for age, gender, and mental health. All four factors relate significantly negatively to the participants’ self-rated health as seen in Table 5. (The participants were asked “In general, how is your health?” and answered on a six-point Likert scale.)

The internal consistency reliability of the entire RSS and subdomains by using Cronbach’s alpha was 0.9359. For the sub-domains (Factor 1–4), the reliability estimates ranged from 0.86 to 0.76.

### Description of new sub-domains

#### Alienation

Compared to the other sub-domains, people experiencing alienation indicate more often that they had been influenced by spirits and spells in the past, and they have the lowest score in present influence. The RSS items corresponding to this sub-domain reflect feelings of hurt, isolation, misunderstanding, and not being happy with the way religious activities are carried out. Alienation seems to be the reaction to past negative experiences of religion and shows itself in following ways: The first priority in life is health and independence and compared to others, and they have the lowest score in putting the relationship with God as the priority. They also have the highest score in “no religious affiliation.” People belonging to this sub-domain also have the least use of herbal supplements see (Table 4).

**Table 4:** External validation of factors: Associations of new RSS sub-domains with alternative measures of religious practice.

Variable	Factor 1 Alienation binary (items’ score average > 3)	Factor 2 Confusion binary (items’ score average > 3)	Factor 3 Conflict/ doubt binary (items’ score average > 3)	Factor 4 Religious zeal binary (items’ score average > 3)
Regression analysis, controlling for age and gender	Coef. (95% CI)	Coef. (95% CI)	Coef. (95% CI)	Coef. (95% CI)
Time spent for religious activities standardized variable	1.2 (0.95, 1.07)	1.22 (0.97, 1.09)	1.24 (0.99, 1.11)	1.44 (1.19, 1.31)
Ordered logistic regression, controlling for age and gender	OR. (95% CI)	OR. (95% CI)	OR. (95% CI)	OR. (95% CI)
God is priority in life (binary)	***1.2** (0.46, 0.74)*	1.23 (0.51, 0.79)	1.43 (0.61, 0.93)	1.56 (0.75, 1.08)
More than one religious affiliation (binary)	4.18 (0.61, 1.6)	4.85 (0.82, 1.99)	3.94 (0.64, 1.58)	2.92 (0.53, 1.24)
I believe that God exist and have no doubts about it (binary)	0.9 (0.4, 0.6)	1.07 (0.49, 0.73)	***0.72** (0.34, 0.5)*	0.9 (0.46, 0.64)

Ordered logistic regression and simple regression analyses, controlled for age and gender. **Italic** and **bold** values are equal to the lowest values and underlined values are equal to highest values. RSS validation study among young people living with HIV in Zimbabwe in 2021.

#### Confusion

The experience of confusion is represented in the highest percentage of having more than one religious affiliation and conflicting beliefs regarding the origin of HIV. Compared to the other sub-domains, people experiencing confusion have the strongest disbelief in the power of spirits, the highest percentage of conspiracy beliefs regarding HIV, and, most importantly, the highest score in “ever stopped ARVs for religious reasons” and negative religious coping. Next to confused religious beliefs, the items under this sub-domain express feelings of insecurity, loss of meaning, rejection, and anger.

**Table 5:** Associations of new RSS sub-domains with self-rated health with six-point Likert scale (1 = excellent health, 6 = very poor health).

Variable	Factor 1 Alienation binary (items’ score average > 3)		Factor 2 Confusion binary (items’ score average > 3)		Factor 3 Conflict/ Doubt binary (items’ score average > 3)		Factor 4 Religious zeal binary (items’ score average > 3)	
Ordered logistic regression, controlling for age and gender	OR. (95% CI)	*p (two-sided)*	OR. (95% CI)	*p (two-sided)*	OR. (95% CI)	*p (two-sided)*	OR. (95% CI)	*p (two-sided)*
Personal health	2.2 (1.14, 1.59)	0.006	1.94 (1.04, 1.42)	0.026	2.1 (1.13, 1.54)	0.006	1.77 (1.04, 1.36)	0.025

Ordered logistic regression analyses, controlled for age and gender. RSS validation study among young people living with HIV in Zimbabwe in 2021.

#### Conflict/doubts

This sub-domain describes intellectual engagement with and questioning of religious, traditional, and spiritual norms, attitudes, and behaviors. There is an element of religious doubt, scoring lowest in the conviction that God exits and using religion in stress handling. Compared to the other domains, this sub-domain scores highest in choosing independence as the priority. Friends as the priority attain the lowest score. Doubt and intellectual questioning or confronting of R/S matters and traditions in a context of religious conformity may lead to the feelings expressed by the items under this domain. They describe existential questions, struggle with the numinous, and a sense of not belonging.

#### Religious zeal

Tijetien states that religious zeal can be a love-like passion and an anger-like emotion that may lead to uncompromised actions. According to her, religious zeal deplores the violation of a religious norm, because religious norms are understood as being of fundamental validity and overall applicability (Tietjen, 2021). The religious zeal sub-domain is confirmed by the lowest score in no religious affiliation, and highest affiliation scores in religious communities tendentially open to spirit interaction (Traditional, Apostolic, Pentecostal, and Muslim). Compared to the other sub-domains, religious zeal has the strongest disagreement with “I do not know whether there is a God or some higher being, and I do not believe there is any way to find out” and the highest percentage of believing in the power of spirits and the use of herbal supplements. It also has the highest percentage in traditional and moralizing views of HIV/AIDS and religious reasons against the use of Western medicine. It scores highest on religion’s involvement in stress handling, using religious positive coping. While these associations describe the “love-like passion”, the RSS items belonging to this sub-domain express the struggle related to the failure of living up to these high religious standards. They describe self-questioning and self-condemnation, feelings of strained relationships with others, God, and the spiritual world, and the loss of ultimate meaning.

## Discussion

Based on the literature review, we expected 20% of the patients to have high R/S struggles. With 16%, the prevalence of considerable-to-strong R/S struggles was less than that hypothesized. Even more, the significant relationship between the prevalence of R/S struggles and perceived personal health needs to be acknowledged. R/S struggles increase the suffering of YPLHIV. In our view, this illustrates the importance of screening for R/S struggles with measures that are culturally sensitive and meaningful.

Since its development in 2014, the RSS has been widely used in and outside the United States. Esperandio’s recent database search found 67 studies that used the RSS or referred to it (Esperandio et al., 2022). Besides her own validation of the RSS in the Brazilian context, the RSS was also validated in Poland (Zarzycka et al., 2018), Iran (Ebrahimi Jamarani et al., 2022), among people with Jewish (Abu-Raiya et al., 2016) and Muslim (Abu-Raiya et al., 2015)) religion in Israel, and in the Czech Republic (Janu et al., n.d.). While the Brazilian, Iranian, Polish, and Jewish samples revealed similar psychometric properties to the original RSS version, the Muslim sample was different, resulting in five sub-domains: three sub-domains identical to the original (Interpersonal, Moral, Ultimate Meaning), one sub-domain with items from the two dimensions Doubt and Divine, and the fifth sub-domain was composed of Demonic items and one item of the Divine domain. The findings of the Czech validation of the RSS suggested a solution with three sub-domains. These were composed of items from the Divine, Interpersonal, and Meaning sub-domains of the original RSS (Janu et al., n.d.).

R/S struggles are prevalent in all these societies and have been linked to poorer life satisfaction, wellbeing, mental health, and, as in our findings, general health. The RSS has been shown to be a valid instrument to measure R/S struggles in different contexts. However, acknowledging the different validation results of the RSS in a non-Christian (Muslim and secular) and African (Zimbabwe) environment demonstrates the importance of adapting the sub-domains to individual cultural and religious contexts.

The limitations of this study are that the RSS was part of a large questionnaire. The application by phone took up to 60 min. Fatigue might have influenced the careful answering of the 26 items. The initial testing of the translation was conducted by the data collector team. External testing was only performed after the data collection and revealed three items that diverted substantially from the original. However, these three items fit well into the sub-domains identified by the EFA. Furthermore, our sample size might not have been big enough for coherent results with SEM and MSA. Finally, it needs to be considered that the participants of this study were all aged between 14 and 24 years and were part of an HIV program. The findings are not to be generalized outside this context.

We therefore suggest that the RSS and the new sub-domains should be tested further among different populations in the sub-Saharan context.

## Conclusion

The RSS proved to be a useful tool in the assessment of R/S struggles among the defined group of YPLHIV in Zimbabwe who participated in this study. R/S struggles are significantly associated with personal health. Thus, care givers can use the RSS to identify potential health risks for YPLHIV. The sub-domains identified by EFA are coherent and culturally relevant. The sub-domains can help to discover the nature of R/S struggles more precisely, and YPLHIV can be supported in a more individualistically, holistically, and culturally relevant manner. It would be helpful to test the RSS and these subdomains in other sub-Saharan population groups. It might well be that the RSS and these adapted sub-dimensions are a new helpful tool, not only to identify R/S struggles but also to develop new, comprehensive, and culturally adapted care and support programs.

## Data availability statement

The raw data supporting the conclusions of this article will be made available by the authors, without undue reservation.

## Ethics statement

The studies involving human participants were reviewed and approved by Medical Research Council Zimbabwe (MRZ/A/2701). Written informed consent to participate in this study was provided by the participants' legal guardian/next of kin.

## Author contributions

UW-G was involved in the conception and design of the study, the analyzing and interpreting of the data, drafted the manuscript, design of the original study protocol, and reviewed the manuscript. SM was involved in the conception and design of the study, the statistical analysis and interpretation of the data, and the editing and revising of the manuscript. AM was involved in the data collection and edited the manuscript. CM was involved in interpreting the data and reviewing the manuscript. NM was responsible to overview the correct implementation of the study protocol and edited the manuscript. MM-M was involved in the overview of the correct implementation of the study protocol and edited the manuscript. All authors contributed to the article and approved the submitted version.

## Funding

Funding was private and with seed money from Swiss Tropical and Public Health Institute.

## Conflict of interest

The authors declare that the research was conducted in the absence of any commercial or financial relationships that could be construed as a potential conflict of interest.

## Publisher’s note

All claims expressed in this article are solely those of the authors and do not necessarily represent those of their affiliated organizations, or those of the publisher, the editors and the reviewers. Any product that may be evaluated in this article, or claim that may be made by its manufacturer, is not guaranteed or endorsed by the publisher.

## Footnotes

^1^ https://www.unicef.org/zimbabwe/hivaids

^2^ https://zvandiri.org/what-we-do/

^3^ https://www.stata.com/capabilities/factor-analysis/

^4^ https://www.stata.com/features/structural-equation-modeling/

^6^ https://www.analysisinn.com/post/kmo-and-bartlett-s-test-of-sphericity/

## Abbreviations

AIDS: Acquired Immune Deficiency Syndrome
BIAS: Belief in Action Scale
CATS: Community Adolescent Treatment Supporters
CFA: Confirmatory Factor Analysis
EFA: Exploratory Factor Analysis
HIV: Human Immunodeficiency Virus
KMO: Kaiser–Meyer–Olkin
ODK: Open Data Kit
MoHCC: Ministry of Health and Child Care
MSA: Mokken Scale Analysis
R/S: Religious/Spiritual
RSS: Religious and Spiritual Struggles Scale
SEM: Structural Equation Modeling
YPLHIV: Young People living with HIV
